# PDZD7 connects the Usher protein complex to the intraflagellar transport machinery

**DOI:** 10.1186/2046-2530-4-S1-P12

**Published:** 2015-07-13

**Authors:** E De Vrieze, L Creemers, N Sorusch, M Gorris, J Van Reeuwijk, S Van Beersum, B Knapp, K Boldt, M Ueffing, R Roepman, E Van Wijk, U Wolfram, H Kremer

**Affiliations:** 1 Department of Otorhinolaryngology, Radboud University Nijmegen Medical Centre, Nijmegen, the Netherlands; 2 Radboud Institute for Molecular Life Sciences, Radboud University Nijmegen, PO Box 9101, 6500 HB Nijmegen, the Netherlands; 3 Department of Cell and Matrix Biology, Institute of Zoology, Johannes Gutenberg University of Mainz, Mainz, Germany; 4 Department of Human Genetics, Radboud University Nijmegen Medical Centre, Nijmegen, the Netherlands; 5 Division of Experimental Ophthalmology and Medical Proteome Center, Centre for Ophthalmology, Eberhard Karls University Tübingen, Tübingen, Germany

## 

Several Usher syndrome (USH)-associated proteins are known to localize to the connecting cilium of photoreceptor cells. The unconventional myosin MYO7A (USH1B) was long accepted as the transport molecule responsible for the ciliary localization of USH proteins. However, based on the typical location of several of the USH proteins along the ciliary axoneme, the involvement of the main ciliary trafficking machinery, intraflagellar transport (IFT), seems apparent.

The USH-associated scaffold protein PDZD7 is known to interact with SANS, Usherin, GPR98 and Whirlin, all of which can be found in the connecting cilium. Here, we report that PDZD7 provides the physical link of the USH-protein network to IFT-complex members. Tandem affinity purification (TAP) studies revealed a potential interaction between PDZD7 and several IFT molecules, including IFT25 and IFT27. TAP analyses of the other USH proteins were negative for IFT proteins, suggesting that this interaction is unique for PDZD7. In addition, a dedicated yeast two-hybrid screen of 200 (predicted) ciliary proteins revealed an interaction between PDZD7 and IFT57. The interaction between PDZD7 and selected IFT subunits was substantiated by co-immunoprecipitations. In accordance with these results, mRFP-tagged PDZD7, expressed in ciliated hTERT-RPE1 cells, localizes not only at the basal body, but also at the axoneme of a subset of cells.

Pending further validation of the interaction between PDZD7 and IFT-B proteins, these first results suggest PDZD7 as a functional connection between USH-proteins and the IFT machinery. Future studies should reveal whether PDZD7 is involved in IFT of USH proteins *in vivo*.

